# *Trametes hirsuta* as an Attractive Biocatalyst for the Preparative Scale Biotransformation of Isosafrole into Piperonal

**DOI:** 10.3390/molecules28083643

**Published:** 2023-04-21

**Authors:** Dawid Hernik, Ewa Szczepańska, Elisabetta Brenna, Katarzyna Patejuk, Teresa Olejniczak, Tomasz Strzała, Filip Boratyński

**Affiliations:** 1 Department of Food Chemistry and Biocatalysis, Wroclaw University of Environmental and Life Sciences, Norwida 25, 50-375 Wrocław, Poland; ewa.szczepanska@upwr.edu.pl (E.S.); teresa.olejniczak@upwr.edu.pl (T.O.); 2 Dipartimento di Chimica, Materiali ed Ingegneria Chimica “Giulio Natta” Politecnico di Milano, Via Mancinelli 7, I-20131 Milan, Italy; mariaelisabetta.brenna@polimi.it; 3 Department of Plant Protection, Wroclaw University of Environmental and Life Sciences, Grunwald Square 24A, 50-363 Wrocław, Poland; katarzyna.patejuk@upwr.edu.pl; 4 Department of Genetics, Wroclaw University of Environmental and Life Sciences, ul. Kozuchowska 7, 51-631 Wrocław, Poland; tomasz.strzala@upwr.edu.pl

**Keywords:** piperonal, isosafrole, alkene cleavage, biotransformation, whole cells, microbial oxidation

## Abstract

Piperonal is a compound of key industrial importance due to its attractive olfactory and biological properties. It has been shown that among the fifty-six various fungal strains tested, the ability to cleave the toxic isosafrole into piperonal through alkene cleavage is mainly found in strains of the genus *Trametes*. Further studies involving strains isolated directly from different environments (decaying wood, fungal fruiting bodies, and healthy plant tissues) allowed the selection of two *Trametes* strains, *T. hirsuta* Th2_2 and *T. hirsuta* d28, as the most effective biocatalysts for the oxidation of isosafrole. The preparative scale of biotransformation with these strains provided 124 mg (conv. 82%, isolated yield 62%) and 101 mg (conv. 69%, isolated yield 50.5%) of piperonal, respectively. Due to the toxic impact of isosafrole on cells, preparative scale processes with *Trametes* strains have not yet been successfully performed and described in the literature.

## 1. Introduction

Piperonal (**1b**), also known as heliotropin (3,4-methylenedioxybenzaldehyde), is an organic compound belonging to aromatic aldehydes. It is a secondary metabolite produced by plants such as heliotrope, vanilla, camphor, violet, and black pepper [1]. Despite its rare occurrence in nature, piperonal has a relevant role in the flavor and fragrance industries. It is reminiscent of vanilla, with a pleasant floral-powder note, and therefore, it is used as an aroma in perfumes, scented candles, detergents, and air fresheners. Additionally, it constitutes a useful intermediate for the synthesis of fine chemicals, such as Tropional^®^ (a commercial fragrance), piperine (a dietary supplement that increases the bioavailability of other compounds in food), α-methyldopa (an antihypertensive), and piribedil (an antiparkinson agent). Furthermore, piperonal (**1b**) can be used for conversion into the psychoactive drug MDMA (3,4-methylenedioxy-*N*-methylamphetamine); therefore, the production and trading of piperonal (**1b**) and its precursors are subject to strict control and regulation in many countries. It is noteworthy that piperonal (**1b**) has been approved for use as a food additive by the U.S. Food and Drug Administration (FDA) and the European Union.

The commercial route of obtaining the piperonal (**1b**) is based on the isomerization of safrole into isosafrole (**1a**) and its subsequent oxidation with chromic acid or ozone, combined with sulfur or a zinc reduction. These processes have a strong environmental impact in terms of the toxicity of the employed chemicals and their high energy consumption for ozone production [2]. Besides the abovementioned method, other synthetic routes have been studied. Lucarelli et al. applied the electrochemical oxidation of piperonyl alcohol using an Au/CeO_2_ catalyst [3]. Alvarez et al. [4] described a process of oxidation of isopropenylbenzenes to the corresponding aldehydes under the influence of microwave radiation with the use of commercial oxidants (PhI(OAc)_2_ on NaY and PhI(OAc)_2_ on Al_2_O_3_). Oppenauer’s oxidation of piperonylic alcohol with different heterogeneous commercial catalysts using paraformaldehyde as a reactant was proposed by Borzatta et al. as an alternative route to obtain piperonal [5].

Due to the growing awareness of consumers regarding the origin of food additives, as well as the growing demand in the industry for piperonal (**1b**), the development of an alternative method for its synthesis is currently being researched. Biotechnological methods constitute the most attractive solution as they are more sustainable and environmentally friendly processes with an emphasis on the prevention of waste generation and avoidance of hazardous compounds. Several methods for piperonal synthesis using biocatalysts have been proposed in the literature. The main substrate used in these methods has been isosafrole (**1a**) [2,6,7,8]. Recently, a chemo-enzymatic three-step procedure for the conversion of isosafrole into piperonal was proposed by Tentori et al. [9]. Additionally, processes for the biotechnological preparation of piperonal, where piperonylic acid and piperonyl alcohol were used as the starting substrates, have been presented in the literature [10,11].

This paper reports a biotransformation process of isosafrole (**1a**) that leads to the production of piperonal (**1b**), a compound of outstanding importance in the fragrance and pharmaceutical industries. A selection of microbial strains exhibiting the desired biocatalytic properties was performed, which resulted in determining strains from the genus *Trametes* as the best biocatalyst agents able to provide piperonal with high conversion and isolation yield. In addition, during the screening process, vicinal diol **1c**, a dihydroxy derivative of isosafrole (**1a**), was obtained as one of the biotransformation products, and it may constitute a novel alternative substrate to produce piperonal (**1b**). This is the first report on the successful application of preparative scale biotransformation using whole cells of Basidiomycetes strains, which allows for obtaining piperonal **1b** from isosafrole **1a** in a considerable amount despite its known inhibitory effect on microbial cells [2].

## 2. Results and Discussion

### 2.1. Screening Scale Biotransformations

**Figure 1:**
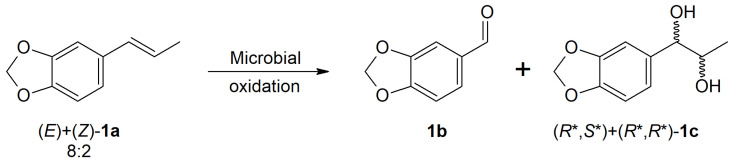
Products of isosafrole (**1a**) biotransformation: piperonal (**1b**) and vicinal diol (**1c**).

Initially, twenty-three different fungal strains were tested for the biotransformation of commercial isosafrole (**1a**), which was used as an 8:2 mixture of (*E*)/(*Z*) diastereoisomers for the desired product (piperonal (**1b**)). These strains were selected from various sources on the basis of literature data, as well as our research group’s experience with the bio-oxidation process on numerous chemical compounds. The vast majority of the tested strains showed no activity towards substrate **1a**, nor did they metabolize it. This is likely related to the toxicity of isosafrole (**1a**) and its growth inhibitory activity towards microorganisms [2]. However, several fungal strains were selected to deliver products **1b** and **1c**, which are shown in Figure 1.

The Table 1 shows the strains that exhibited peroxidase activity towards isosafrole (**1a**) in the preliminary screening studies. The presence of piperonal (**1b**) was detected in extracts from the cultures of *Trametes versicolor* AM536, *T. hirsuta* d28, and *Mortierella isabelina* AM212. It was noteworthy that in the extracts collected from *Piptoporus betulinus* AM40 and *Laetiporus sulphurens* AM515, vicinal diol **1c** was observed as a sole product.

Vicinal diol **1c** could be considered an intermediate product for obtaining piperonal (**1b**) in a more effective way. Given the toxicity of isosafrole (**1a**) in relation to microorganisms, the use of diol **1c** as a starting material might be a superior approach for obtaining piperonal (**1b**). Our research team is currently investigating this alternative route by using bacteria known for their oxidizing activity after producing diol **1c** from isosafrole by chemical synthesis. The microbiological transformation of diol **1c** into piperonal could then be considered as an alternative to the MnO_2_ oxidative cleavage of **1c** described by Tentori et al. [9].

Santos et al. tested several strains of fungi (*Aspergillus flavus*, *A. niger*, and *Cladosporium sphaerospermum*) and bacteria (*Pseudomonas aeruginosa* and *P. putida*) for the conversion of isosafrole (**1a**) to piperonal (**1b**) [6]. The highest conversion (only 9.8%) was achieved with *C. sphaerospermum* after 7 days of biotransformation. Similar research was conducted on a broader screening of several dozen strains of fungi, yeasts, and bacteria which primarily belonged to the genus *Saccharomyces*, *SAspergillus*, *SFusarium*, and *Trichoderma*. *SPeacilomyces variotii* was selected as the most promising biocatalyst as the filamentous fungi showed better oxidative activity towards compound **1a** than the other microorganisms. As a result, only 20% conversion was obtained after 68 h of using a *P. variotii* post-culture medium containing extracellular peroxidases [2]. However, no isolation yields were reported in the aforementioned studies. Zhao et al. screened bacteria isolated from the soil for the bioconversion of isosafrole (**1a**) to piperonal (**1b**). For this purpose, a color screening method was used. Aromatic aldehydes were detected by a reaction with 2,4-dinitrophenylhydrazine (DNPH) to produce the corresponding colored hydrazone derivative (in the form of a red precipitate), with positive correlation between the amount of precipitate and the concentration of piperonal (**1b**). One of the tested strains, identified as *Serratia liquefaciens*, provided the highest piperonal (**1b**) concentration at the level of 282.32 mg/L with a molar efficiency of 35.8% after the optimization of culture conditions [7]. A fungus of the genus *Trametes* proved to be a very interesting biocatalyst for obtaining piperonal (**1b**) as in its culture the product showed higher stability and was not metabolized by cells. The research conducted by Lara et al. confirmed the ability of these fungi to produce the peroxidases responsible for biocatalytic alkene cleavage in the *trans*-anethol, leading to the production of the corresponding aldehyde [8]. They also attempted to convert isosafrole (**1a**); however, only 4% of piperonal (**1b**) was obtained.

### 2.2. The Influence of Carbon and Nitrogen Source on Biotransformation

Due to the unsatisfying amount of piperonal (**1b**) (14%) obtained in the culture of *T. versicolor* AM536, it was decided to determine whether changing the carbon and nitrogen sources in the biotransformation media would improve the oxidation efficiency of the isosafrole (**1a**) to piperonal (**1b**). Preliminary tests were carried out using *T. versicolor* AM536 where the glucose in Saboraud medium was replaced with various sugars such as fructose, mannose, ribose, starch, and galactose. Several attempts were also made to replace the peptone by using alternative sources of organic nitrogen, such as casein hydrolysate, meat, potatoes and soy peptones, and lactalbumin hydrolysate. These studies showed that when in a modified Sabouraud medium with ribose and peptone as sources of carbon and nitrogen, respectively, the amount of piperonal (**1b**) obtained increased to 18% (Table 2). When the carbon source was glucose and the nitrogen source was casein peptone, it reached 33%.

Subsequently, the biotransformations were performed with *T. versicolor* AM536 and *T. hirsuta* d28 by increasing the amount of added substrate (50 mg) to the growing biomass and using various selected combinations of the optimal carbon and nitrogen sources (Table 3).

Unexpectedly, it appeared that with both strains, better results were obtained in the standard Sabouraud medium. Glucose as a carbon source had a positive effect on the conversion of isosafrole (**1a**) to piperonal (**1b**) while the nitrogen source did not have a significant impact on the biotransformation. The highest conversion of **1a** was reported for the *T. hirsuta* d28 culture, where 43% of aldehyde **1b** was obtained.

Due to the unsatisfactory amount of biocatalysts selected during the screening study (out of 23 tested strains, only 5 showed activity against isosafrole **1a**) (Table 1), it was decided to extend the search and examine the abilities of other fungi for the biooxidation of an unsaturated bond in substrate **1a**. Due to the kindness of researchers from the Department of Plant Protection, various strains of fungi isolated directly from different environments (decaying wood, fungal fruiting bodies, and healthy plant tissues) were provided to us for further tests (see Materials and Methods, Section 3.2). The study confirmed that the vast majority of the tested strains did not bring the expected results and did not exhibit the ability to transform isosafrole (**1a**). The exceptions were the strains of the species *Trametes hirsuta*, which turned out to be the only biocatalysts that showed activity towards substrate **1a** among all the tested strains (Table 4). The literature data has indicated that strains of the *Trametes* genus are well-known for having alkene cleaving properties in a wide range of chemical compounds [12,13,14,15,16,17,18,19,20,21]. In particular, very promising results were obtained during the process using *T. hirsuta* Th2_2 as a biocatalyst, where 38% of piperonal (**1b**) was obtained after 14 days of biotransformation. The other tested strains of *T. hirsuta* delivered only diol **1c**.

### 2.3. Preparative Biotransformations of Isosafrole (***1a***) to Piperonal (***1b***)

Considering the most promising biocatalysts for producing piperonal (**1b**) (*T. hirsuta* Th2_2 and *T. hirsuta* d28), it was decided to perform preparative scale biotransformations. In the transformation conducted with *T. hirsuta* Th2_2 using 100 mg of substrate **1a**, after 11 days, the conversion of isosafrole (**1a**) reached 95%, where 38% of piperonal (**1b**) and 34% of vicinal diol (**1c**) were recorded. When *T. hirsuta* d28 was employed, 41% of product **1b** and 54% of product **1c** were obtained. The latter was subsequently isolated with a yield of 49%. The isolated compound was a 3:1 mixture of (R*,S*)- and (R*,R*)-diols **1c**, which were assigned based on the spectroscopic data. Our results are in accordance with the fact that isosafrole (**1a**), which was submitted for biotransformation, is a commercial mixture of (*E*)/(*Z*) diastereoisomers. The relative configuration of the two diastereoisomers of diol (**1c**) was established on the basis of the fact that the starting isosafrole was enriched in the (*E*)-stereoisomer and that the hydrolytic epoxide cleavage occurred with an anti-mechanism [9].

Despite the use of a fairly high amount of isosafrole (**1a**), no negative effect of this compound on the cells of the tested fungi was observed, and as a result, it was decided to conduct the process by adding twice as much substrate **1a** (200 mg) to the *T. hirsuta* d28 and *T. hirsuta* Th2_2 cultures. The process was controlled at the appropriate intervals and stopped on the 11th day when a satisfactory conversion of isosafrole (**1a**) was obtained and no increase in product **1b** over that time period had been observed. After product purification, piperonal (**1b**) was obtained from the *T. hirsuta* Th2_2 and *T. hirsuta* d28 cultures with isolation yields of 62% (124 mg) and 50.5% (101 mg), respectively (Table 5).

It is worth mentioning that the use of whole microbial cells in the piperonal (**1b**) synthesis from isosafrole (**1a**) has not yet been successfully accomplished in the preparative process. Currently known methods involve the use of purified enzymes or *Escherichia coli* bacteria which, as a result of genetic modifications, express the enzymes leading to the production of the aldehyde **1b**. Schwendenwein et al. proposed an alternative method of obtaining piperonal (**1b**) by the enzymatic reduction of piperonylic acid using carboxylate reductase (CAR) from *Neurospora crassa* expressed in *E. coli*. The preparative scale was conducted in two 2 L flasks (200 mL of medium), adding 0.99 g of piperonylic acid to each flask. They obtained 100% piperonal with a 92% isolation yield (1.66 g) [10]. The purified aryl-alcohol oxidase PeAAO2 from *Pleurotus eryngii* P34 was used to obtain piperonal (**1b**) in the preparative scale from piperonyl alcohol as a substrate [11]. The scale-up biotransformation was conducted in two 100 mL flasks containing 10 mL of potassium phosphate buffer with 0.5 µM of purified enzyme and catalase. This approach allowed for achieving a 95% of conversion within 3 h, and the final product was extracted with an 85% yield (244.6 mg of piperonal). The biosynthesis of piperonal (**1b**) by engineered *E. coli* co-expressing two enzymes (*trans*-anethole oxygenase and formate dehydrogenase) was proposed as a novel method by Wen et al. [22]. The final concentration of product **1b** was 19.45 g/L, and the maximum yield and space-time yield of the isosafrole (**1a**) bioconversion reached 96.02% and 3.89 g/L/h, respectively. The table below (Table 6) summarizes the approaches to piperonal (**1b**) preparation published so far using various types of biocatalysts. Both preparative and screening scale processes are included.

The approaches discussed above are undoubtedly attractive solutions that allow obtaining piperonal (**1b**) in considerable amounts. The use of biocatalysts in the form of genetically modified microorganisms, as well as purified enzymes, allowed the researchers to solve the problem of the toxic effect of isosafrole (**1a**) on microbial cells, which was the main limiting factor described in studies conducted by other scientists. As Table 6 indicates, the approaches constituting the application of fungal whole cells for the biotransformation of isosafrole (**1a**) have not been successfully performed in a preparative scale. The method we have proposed includes the use of a biocatalyst in the form of whole cells; therefore, time-consuming and expensive procedures such as an enzyme purification or the use of genetically modified bacterial expression systems can be omitted.

### 2.4. Species Identification

The Th2_2 and d28 strains sequencing showed two different DNA haplotypes (NCBI accession numbers OQ608086 and OQ608087, respectively). Blast analyses showed more than 99% identity to more than one *Trametes* species, and thus, exact species identification with that method was not possible. Phylogenetic trees (see Supplementary Materials, Figure S10) showed the same topology, though with minor differences for BA (*Bayesian analysis*) and ML (maximum likelihood), and we grouped both analyzed sequences with *T. hirsuta* representatives. The node connecting the Th2_2 and d28 sequences with other *T. hirsuta* samples had a high Bayesian probability (100%) and a high a-LRT bootstrap value (94%), suggesting that both analyzed samples belonged to the *T. hirsuta* species.

## 3. Materials and Methods

### 3.1. Materials

The isosafrole as an 8:2 mixture of (*E*)/(*Z*) diastereoisomers was purchased from Zentek s.r.l. (Milan, Italy). All chemicals used for the media preparation were purchased from Sigma-Aldrich Chemical Co., St. Louis, MO, USA. The solvents used for the extractions and the GC analysis were purchased from Stanlab (Lublin, Poland).

### 3.2. Microorganisms

The strains *Absidia cylindrospora* AM336, *Armillaria mellea* AM296, *A. mellea* AM461, *Aspergillus ochraceus* AM456, *Chaetomium* sp. AM432, *Fusarium culmorum* AM282, *F. equiseti* AM22, *F. oxysporum* AM21, *Inonotus radiates* AM70, *Laetiporus sulphurens* AM498, *Laetiporus sulphurens* AM515, *Mortierella isabelina* AM212, *Papularia rosea* AM17, *Pholiota aurivella* AM522, *Piptoporus betulinus* AM40, *P. betulinus* AM57, *Pleurotus ostreatus* AM482, *Poria placenta* AM36, *P. placenta* AM38, and *Trametes versicolor* AM536 were obtained from the microbial collection of the Department of Food Chemistry and Biocatalysis at the Wroclaw University of Environmental and Life Sciences (AM). *Agrocybe aegerita* DSM 22459 and *Pleurotus sapidus* DSM 8266 were purchased from the German Collection of Microorganisms and Cell Cultures (DSMZ) in Braunschweig. *Pycnoporus cinnabarinus* CBS 353.63 was purchased from the Westerdijk Fungal Biodiversity Institute (CBS) in Utrecht (The Netherlands). The fungal strains were maintained at 4 °C on Sabouraud (SB) agar slants containing peptone (10 g), glucose (30 g), and agar (15 g) dissolved in water (1 L) at a pH of 5.5, and then they were transferred into conical flasks with SB medium.

Many strains used for the screening were obtained directly for this study from different environments (decaying wood, fungal fruiting bodies, and healthy plant tissues) and deposited in the microbial collection of the Department of Plant Protection at the Wroclaw University of Environmental and Life Sciences. Based on their macro- and microscopic features, they were labeled as: *Trametes hirsuta*: d28, TH_ID007, TH1_1, TH2_2, TH5_2, TH5_3, TH3_2, TH5_1, TH1/2, TH3_4, and TH3_1; *Marasmius cohaerens* ID636_Mar_cohaerens; *Corpinus* sp.: 41_Corp and BS_Corp1/1; *Peniophora quercina*: Perenochaeta_3, Perenochaeta_1, and Perenochaeta_2; *Trichoderma* sp. WR1_1 and *Aureobasidium pullulans* Aureo; *Chaetopsis* sp. *Chaetopsis*; *Mucor* sp. BS_skórnik2 and *Penicillium* sp. BSwr6_4; *Daedalea quercina* BS_Gmatwek2/3; and unidentified: WR4_1, WR4_3, WR3_4, WR3_3, WR3_1, WR3_2, WR4_2, WR2_6, TH3_1, Th4, and wr2_6.

### 3.3. Fungi Isolation Methods

During the fieldwork (2021–2022), samples were collected in sterile envelopes and transported to the laboratory where the isolates underwent mycological analysis. To obtain pure fungal strains from the environmental samples for the screening, different methods of isolation were used.

Most strains were isolated from decaying wood tissues (white-rot) in accordance with the methodology proposed by Arhipova et al. [23] using PDA medium (potato dextrose agar, Biocorp). They were isolated from fir’s wood, Gorce National Park, Poland (d28); beech wood, Złoty Stok mine region, Poland (TH2_2, TH3_2, Perenochaeta_1, Perenochaeta_2, TH5_1, TH1/2, TH3_4, TH3_1, WR1_1, WR4_1, WR4_3, WR3_4, WR3_3, WR3_1, WR3_2, WR4_2, WR2_6, TH5_2, TH3_1, Th4, and wr2_6); spruce wood, Jetřichovice village, region of Bohemian Switzerland, Czech Republic (BS_skórnik2, BSwr6_4, and BS_Corp1/1); and sycamore wood, Jetřichovice village, region of Bohemian Switzerland, Czech Republic (BS_Gmatwek2/3).

Some strains were isolated from fungal fruiting bodies. In this method, small pieces (3 mm × 3 mm) of the mycelium obtained from the inner part of fungal cup (to avoid environmental cross-contamination) were placed on PDA medium. They were isolated from *T. hirsuta* cup, Racibórz forest, Poland (TH_ID007); *T. hirsuta* cup, Złoty Stok mine region, Poland (TH1_1 and TH5_3); *P. quercina* fruiting body, Złoty Stok mine region, Poland (Perenochaeta_3); and *M. cohaerens* cup, Wigry National Park, Poland (ID636_Mar_cohaerens).

Three strains were obtained from living plant tissues with no visible symptoms of pathogenesis or decay. Two fungal strains (41_Corp and *Chaetopsis*) were isolated from the flowers of *Impatiens glandulifera*, which were growing in the Kraków municipality. One strain (Aureo) was isolated from a plum’s leaf from the Wrocław municipality. A rinsing method using Martin medium (BTL Ltd., Potomac, MD, USA) was used to obtain all these strains.

In every method, growing colonies of fungi were passaged, and then clean mycelium was obtained by the method of monospore cultures.

### 3.4. Molecular Identification of Fungal Strains

To confirm the species identity of the two most promising biocatalysts that produced piperonal (the *T. hirsuta* Th2_2 and *T. hirsuta* d28 strains), molecular identification was performed. DNA was isolated from the culture growths on slants with TSA medium. Mycelial fragments were placed in 1.5 mL tubes, together with 500 μL of TE buffer, and heated in a microwave at 600 W for two minutes. Next, the tubes were centrifuged at ~6000 g for two minutes and the supernatant from each tube was transferred onto an isolation column from a Sherlock AX isolation kit (A&A Biotechnology, Gdańsk, Poland). Finally, DNA was extracted according to the producer’s manual and suspended in TE buffer.

The isolated DNA was quantified with a Qubit 4 Fluorometer and diluted to unify the concentration among the samples. An internal transcribed fragment was amplified with the primers ITS4 and ITS5 with the following conditions: initial denaturation at 95 °C—2 min; 35 cycles at 95 °C—60 s; 55 °C—60 s; 72 °C—90 s; and final elongation at 72 °C—10 min. The PCR products were verified with agarose gel (1%) electrophoresis, cleaned with an Eppic enzymatic cleanup kit (A&A Biotechnology, Poland), and, finally, sequenced in both directions using an Applied Biosystems 3730 XL DNA analyzer in Genomed S.A.

After sequencing, both strands of each sample were aligned using Bioedit 7.2.5 software [24] in order to obtain consensus sequences. Both sequences obtained in this way were used in Blast [25] and implemented in the Genbank database (http://www.ncbi.nlm.nih.gov, accessed on 9 March 2023) to search for homologues. Next, phylogenetic analysis was performed using the PolyPeet ITS database used previously in the phylogenetic study of *Trametes* sp. [26]. The trees were created with both the Bayesian and maximum likelihood approaches. MrBayes 3.2.7a [27] was used to estimate the Bayesian trees using the SYM + G + I substitution model, which was chosen as the best-fit model with jModelTest 2.1.10 [28]. The MrBayes analysis consisted of two independent runs (each with four chains) starting from random trees. The trees were sampled every 100th generation for 10,000,000 generations (with 25% burn-in) until the average standard deviation of split frequencies was stabilized at below 0.01 for all trees used to construct the consensus tree. IQTree [29] was used to estimate the maximum likelihood tree with the SYM + G + I model and a Shimodara–Hasegawa-like approximate likelihood ratio test (SH-aLRT) [30] as the tree branch support.

### 3.5. Screening Scale Biotransformations

The biotransformations with the fungal strains were carried out in 250 mL Erlenmayer flasks with 75 mL of liquid culture medium. The flasks were sterilized at 121 °C at a pressure of 1 atm (15 min) and the medium was inoculated with 0.5 mL of pre-prepared fungal cultures under a laminar chamber under sterile conditions. Then, the cultures were shaken at 150 rpm for 5–7 days at room temperature. After the growth of the biomasses, 15 mg (460 mM) of substrate (**1a**) dissolved in 0.2 mL of DMSO was added to the cultures.

### 3.6. Modification of Culture Media Composition

During the study, the impact of various carbon and nitrogen sources on the process was examined with a strain T. versicolor AM536. The biotransformations were carried out in 250 mL Erlenmayer flasks with 75 mL of modified Sabouraud medium where the glucose was replaced by sugars such as fructose, mannose, ribose, starch, and galactose. Instead of peptone, casein hydrolysate, meat peptone, potatoes peptone, soy peptone, and lactalbumin hydrolysate were used. After the growth of the biomasses, 15 mg of substrate (**1a**) dissolved in 0.2 mL of DMSO was added to the cultures. In further studies, the *T. hirsuta* d28 and *T. hirsuta* Th2_2 strains were used, where Sabouraud medium and its modifications (glucose/casein hydrolysate, ribose/peptone, and ribose/casein hydrolysate) were used. During this experiment, 50 mg of substrate (**1a**) dissolved in 0.2 mL of DMSO was added to the culture. The experiments were conducted in duplicates.

### 3.7. Extraction Procedure

To observe the progress of the biotransformation, samples (3 mL) of the reaction mixtures were taken after 7 and 14 days. The aqueous phase was acidified with 0.1 M HCl to pH = 3, salted out with NaCl, and extracted with ethyl acetate (1.5 mL), followed by centrifugation (4000 rpm/3100× *g*, 10 min, 4 °C). The extract was dried over anhydrous magnesium sulfate, filtered, concentrated by rotary evaporator, and analyzed by the GC method.

### 3.8. Preparative Biotransformations

Five-hundred milliliters of Sabouraud medium were placed in 2000 mL flasks and sterilized at 121 °C for 15 min. The medium was inoculated with 50 mL of preprepared cultures of the microorganisms. The flasks with fungal cultures were stored for 7 days at 25 °C and shaken at 150 rpm. After this time, 100 mg or 200 mg of isosafrole dissolved in 5 mL of DMSO was added into the culture and the process was continued at 25 °C and 150 rpm. Samples were extracted after 11 days and observed by GC to estimate the progress of the biotransformation. Subsequently, the products were then extracted three times with 50 mL of ethyl acetate, collected, dehydrated by anhydrous MgSO_4_, and the organic solvent was evaporated under reduced pressure. The samples prepared in this way were then purified using a puriFlash apparatus with hexane:ethyl acetate 4:1 (*v*/*v*). The fractions collected in this way were verified using the GC method, and finally, only those containing the pure product were combined.

### 3.9. Analysis Procedure

Gas chromatography analysis (GC, FID, with the carrier gas H_2_) was carried out on an Agilent Technologies 6890 N (GC System, Santa Clara, CA, USA) with use of a column HP-5 (30 m × 0.32 mm × 0.25 μm, Santa Clara, CA, USA) according the following temperature program: 70 °C and 300 °C (30 °C/min) (1 min). Samples (1 μL) were injected with split 20:1, and the flow of the carrying gas was 1 mL/min. The total run time was 9.8 min. Retention times were established as follow: t_R_ = 4.19 min for piperonal (**1b**), t_R_ = 4.37 min for isosafrole (**1a**), and t_R_ = 5.58 min for the (1R*,2S*)-**1c** and 5.62 min for the (1R*,2R*)-**1c** vicinal diols. GC/MS analyses were performed using an HP-5MS column (30 m × 0.25 mm × 0.25 μm) from Agilent Technologies Italia S.p.A. (Cernusco sul Naviglio, Italy). The following temperature program was employed: 60 °C (1 min), 150 °C (6 °C/min) (1 min), and 280 °C (12 °C/min) (5 min). The total run time was 32 min. The structures of the compounds were confirmed on the basis of ^1^H NMR and ^13^C NMR, which were recorded for the CDCl_3_ solutions on a Bruker Avance DRX 600 (600 MHz) spectrometer (Billerica, MA, USA).

Separation of the biotranformation products during flash chromatography were controlled by thin layer chromatography (TLC) using aluminum foil plates coated with silica gel. The compounds were detected by spraying the plates with 1% Ce(SO_4_)_2_ and 2% H_3_[P(Mo_3_O_10_)_4_] in 10% H_2_SO_4_.

The NMR spectra and GC-MS chromatograms (see Supplementary Materials, Figures S1–S9) of the obtained products were as follows:

Piperonal (**1b**) ^1^H NMR (CDCl_3_, 600 MHz): δ = 9.79 (s, 1H, *CHO*), 7.40 (dd, 2H, *J* = 7.9 Hz and 1.5 Hz, *Ar–H*), 7.32 (d, 1H, *J* = 1.5 Hz, *Ar–H*), 6.91 (d, 1H, *J* = 7.9 Hz, *Ar–H*), 6.06 (s, 2H; *CH_2_*); ^13^C NMR (100 MHz, CDCl_3_): δ = 190.4, 153.2, 148.8, 131.9, 128.8, 108.4, 106.9, 102.2; and GC/MS (EI) tr = 15.26 min: *m*/*z* (%) = 149 (M+ −1, 100), 121 (40), 91 (12), 63 (25).

(1R*,2S*)-1-(Benzo[1,3]dioxol-5-yl)propane-1,2-diol ((1R*,2S*)-**1c**) ^1^H NMR (CDCl_3_, 600 MHz): δ = 6.86–6.84 (m, 1H, *Ar–H*), 6.81–6.76 (m, 2H, *Ar–H*), 5.96 (s, 2H, *CH_2_*), 4.28 (d, 1H, *J* = 7.5 Hz, *CHOH*), 3.80 (m, 1H, *CHOH*), 2.22 (s, 2H; 2x*OH*), 1.03 (d, 3H, *J* = 6.3 Hz, *CH_3_*); ^13^C NMR (CDCl_3_, 100 MHz): δ = 147.91, 147.5, 135.1, 120.5, 108.3, 107.2, 101.1, 79.4, 72.3, 18.9; and GC/MS (EI) tr = 21.23 min: *m*/*z* (%) = 196 (M+, 16), 178 (8), 162 (8), 151 (100), 135 (25), 123 (25).

(1R*,2R*)-1-(Benzo[1,3]dioxol-5-yl)propane-1,2-diol ((1R*,2R*)-**1c**) ^1^H NMR (CDCl_3_, 600 MHz): δ = 6.90–6.89 (m, 1H, *Ar–H*), 6.80–6.78 (m, 2H, *Ar–H*), 5.95 (s, 2H, *CH_2_*), 4.56 (d, 1H, *J* = 4.6 Hz, *CHOH*), 3.95 (m, 1H, *CHOH*), 2.22 (s, 2H; 2x*OH*), 1.10 (d, 3H, *J* = 6.4 Hz, *CH_3_*); ^13^C NMR (CDCl_3_, 100 MHz): δ = 147.87, 147.3, 134.4, 120.2, 108.2, 107.1, 101.2, 77.5, 71.3, 17.6; and GC/MS (EI) tr = 21.31 min: *m*/*z* (%) = 196 (M+, 16), 178 (8), 162 (8), 151 (100), 135 (25), 123 (25).

## 4. Conclusions

In this study, a preliminary screening was performed on fifty-six fungal strains. Only a few of them showed the ability to convert isosafrole (**1a**). Of these, the *Trametes* strains were the most promising biocatalysts. Due to literature reports on the ability of *Trametes* to perform biooxidation on a wide range of compounds, it was decided to extend the screening to include strains from different *Trametes* species. The most efficient biocatalyst appeared to be *T. hirsuta* Th2_2, which provided piperonal (**1b**) with a 62% isolation yield under the preparative biotransformation scale. It is worth mentioning that there are no literature reports on the production of piperonal (**1b**) from isosafrole (**1a**) on a preparative scale using whole cells of fungi. Known methods involve the use of purified enzymes or genetically modified bacteria *E. coli*. Therefore, due to the simplicity of the process, it is important to continue research on this strain to improve its capacity.

## Figures and Tables

**Table 1:** Results of the preliminary screening of strains with peroxidase activity in relation to isosafrole (**1a**) (percentage values according to GC).

Strain	Time (Days)	Substrate (%) 1a	Products (%)	
			1b	1c
*Laetiporus sulphurens* AM515	7	0	0	100
	14	0	0	100
*Mortierella isabelina* AM212	7	100	0	0
	14	87	13	0
*Piptoporus betulinus* AM40	7	23	0	77
	14	17	0	83
*Trametes versicolor* AM536	7	63	10	27
	14	36	14	50
*Trametes hirsuta* d28	7	79	8	13
	14	42	10	48

**Table 2:** Biotransformation of isosafrole (**1a**) by the *Trametes versicolor* AM536 strain in various carbon and nitrogen sources.

Source		Time (Days)	Substrate (%) 1a	Products (%)	
Carbon	Nitrogen			1b	1c
Fructose	Peptone	7	44	3	53
		14	31	3	66
Mannose		7	38	3	59
		14	29	3	68
Ribose		7	33	14	53
		14	18	18	64
Starch		7	52	2	46
		14	34	4	62
Galactose		7	62	0	38
		14	14	0	86
Glucose	Casein hydrolysate	7	19	33	48
		14	7	28	65
	Lactalbumin hydrolysate	7	17	10	73
		14	17	10	73
	Meat peptone	7	51	15	34
		14	61	14	25
	Potatoes peptone	7	83	3	14
		14	65	4	31
	Soy peptone	7	51	3	46
		14	32	4	64

**Table 3:** Biotransformation of isosafrole (**1a**) by *Trametes* strains in selected carbon and nitrogen sources (percentage values according to the GC as the mean value of two replicates).

Strain	Source		Time (Days)	Substrate (%) 1a	Products (%)	
	Carbon	Nitrogen			1b	1c
*Trametes versicolor* AM536	Glucose	Peptone	7	40	20	40
			14	34	24	42
	Ribose	Peptone	7	72	0	28
			14	53	16	31
	Ribose	Casein hydrolysate	7	57	5	38
			14	45	16	39
	Glucose	Casein hydrolysate	7	47	13	40
			14	32	20	48
*Trametes hirsuta* d28	Glucose	Peptone	7	33	35	32
			14	11	43	46
	Ribose	Peptone	7	100	0	0
			14	100	0	0
	Ribose	Casein hydrolysate	7	100	0	0
			14	100	0	0
	Glucose	Casein hydrolysate	7	17	32	51
			14	10	33	57

**Table 4:** Biotransformation of isosafrole (**1a**) by *Trametes hirsuta* strains (percentage values according to the GC).

Strain	Time (Days)	Substrate (%) 1a	Products (%)	
			1b	1c
*T. hirsuta* Th1_1	7	68	0	32
	14	66	0	34
*T. hirsuta* Th2_2	7	24	23	53
	14	8	38	54
*T. hirsuta* Th5_2	7	36	0	64
	14	22	0	78
*T. hirsuta* Th5_3	7	33	0	67
	14	34	0	66

**Table 5:** Summary of the biotransformation process on a preparative scale with 200 mg of isosafrole (**1a**) after 11 days (percentage values according to the GC).

Strain	Substrate (%) 1a	Products (%)		Isolation Yield of 1b (%)	Amount of 1b Obtained (mg)
		1b	1c		
*T. hirsuta* Th2_2	11	82	7	62	124
*T. hirsuta* d28	25	69	6	50.5	101

**Table 6:** Juxtaposition of the biotechnological approaches for obtaining piperonal (**1b**).

Biotransformation Approach	Biocatalysts	Substrate	Conversion (%)	Obtained Amount	Ref.
Whole cells	*Trametes hirsuta*	Isosafrole	4	np *	[8]
Whole cells	*Cladosporium sphaerospermum*	Isosafrole	9.8	np *	[6]
Post-culture medium	*Paecilomyces variotii*	Isosafrole	20	np *	[2]
Whole cells	*Serratia liquefaciens*	Isosafrole	38.5	282.32 mg/L **	[7]
GMO co-expressing system	*Escherichia coli (FDH* and *TAO_3G2_)*	Isosafrole	96.02	19.45 g/L	[22]
Purified enzyme	POAA2 from *Pleurotus eryngii* P34	Piperonylic alcohol	95	244.6 mg	[11]
GMO co-expressing system	*E. coli* (CAR from *Neurospora crassa*)	Piperonylic acid	100	1.66 g ***	[10]

* np—information not provided; ** results obtained after applying DOE technique (RSM); *** reaction performed in two 2 L flasks (200 mL of medium with 0.99 g of substrate each).

## Data Availability

The data presented in this study are available on request from the corresponding author.

## Supplementary Materials

The following supporting information can be downloaded at: https://www.mdpi.com/article/10.3390/molecules28083643/s1, Figure S1: Chromatogram of the preparative scale biotransformation of isosafrole (**1a**) to piperonal (**1b**) with *T. hirsuta* d28 after 11 days; Figure S2: Chromatogram of the preparative scale biotransformation of isosafrole (**1a**) to piperonal (**1b**) with *T. hirsuta* TH2_2 after 11 days; Figure S3: H^1^ NMR spectrum of piperonal (**1b**); Figure S4: C^13^ NMR spectrum of piperonal (**1b**); Figure S5: GC/MS chromatogram of piperonal (**1b**); Figure S6: H^1^NMR spectra of (1*R**,2*S**) and (1*R**,2*R**)-1-(benzo[1,3]dioxol-5-yl)propane-1,2-diol (**1c**); Figure S7: C^13^ NMR spectra of (1*R**,2*S**) and (1*R**,2*R**)-1-(benzo[1,3]dioxol-5-yl)propane-1,2-diol (**1c**); Figure S8: GC/MS chromatogram of (1*R**,2*S**) and (1*R**,2*R**)-1-(benzo[1,3]dioxol-5-yl)propane-1,2-diol (**1c**); Figure S9: GC/MS chromatogram of (1*R**,2*S**) and (1*R**,2*R**)-1-(benzo[1,3]dioxol-5-yl)propane-1,2-diol (**1c**); Figure S10: Bayesian phylogenetic tree of the PolyPeet ITS database sequences of *Trametes* (and other species), along with two sequences from the strains d28 and Th2_2 described in this manuscript. Numbers along the nodes are the posterior probabilities of the nodes (BA) and maximum likelihood bootstrap values (ML) (values below 0.7 are not shown (-)). The *Trametes hirsuta* clade is marked with a red line and both analyzed samples are marked with red stars.

## Sample Availability

Samples of the compounds are not available from the authors.

## References

[1] Rachwalik R. (2018). Technologie Wybranych Związków Zapachowych.

[2] Santos A.S., Pereira N., da Silva I.M., Sarquis M.I.M., Antunes O.A.C. (2004). Peroxidase Catalyzed Microbiological Oxidation of Isosafrol into Piperonal. Process Biochem..

[3] Lucarelli C., Lolli A., Giugni A., Grazia L., Albonetti S., Monticelli D., Vaccari A. (2017). Efficient and Ecofriendly Route for the Solvent-Free Synthesis of Piperonal and Aromatic Aldehydes Using Au/CeO_2_ Catalyst. Appl. Catal. B.

[4] Alvarez H.M., Barbosa D.P., Fricks A.T., Aranda D.A.G., Valdés R.H., Antunes O.A.C. (2006). Production of Piperonal, Vanillin, and p-Anisaldehyde via Solventless Supported Iodobenzene Diacetate Oxidation of Isosafrol, Isoeugenol, and Anethol under Microwave Irradiation. Org. Process. Res. Dev..

[5] Borzatta V., Capparella E., Chiappino R., Impalà D., Poluzzi E., Vaccari A. (2009). Oppenauer’s Oxidation by Paraformaldehyde of Piperonyl Alcohol to Heliotropine. Catal. Today.

[6] Santos A.S., Pereira N.J., da Silva I.I., Sarquis M.I., Antunes O.A.C. (2003). Microbiologic Oxidation of Isosafrole into Piperonal. Appl. Biochem. Biotechnol..

[7] Zhao M., Zheng P., Chen P., Liu S. (2017). Biosynthesis of Heliotropin by a Novel Strain of *Serratia liquefaciens*. Appl. Biochem. Biotechnol..

[8] Lara M., Mutti F.G., Glueck S.M., Kroutil W. (2008). Biocatalytic Cleavage of Alkenes with O_2_ and *Trametes hirsuta* G FCC 047. Eur. J. Org. Chem..

[9] Tentori F., Brenna E., Ferrari C., Gatti F.G., Ghezzi M.C., Parmeggiani F. (2021). Chemo-Enzymatic Oxidative Cleavage of Isosafrole for the Synthesis of Piperonal. React. Chem. Eng..

[10] Schwendenwein D., Fiume G., Weber H., Rudroff F., Winkler M. (2016). Selective Enzymatic Transformation to Aldehydes in Vivo by Fungal Carboxylate Reductase from *Neurospora crassa*. Adv. Synth. Catal..

[11] Jankowski N., Koschorreck K., Urlacher V.B. (2022). Aryl-Alcohol-Oxidase-Mediated Synthesis of Piperonal and Other Valuable Aldehydes. Adv. Synth. Catal..

[12] Mang H., Gross J., Lara M., Goessler C., Schoemaker H.E., Guebitz G.M., Kroutil W. (2007). Optimization of a Biocatalytic Single-Step Alkene Cleavage of Aryl Alkenes. Tetrahedron.

[13] Kurlemann N., Lara M., Pohl M., Kroutil W., Liese A. (2009). Asymmetric Synthesis of Chiral 2-Hydroxy Ketones by Coupled Biocatalytic Alkene Oxidation and C–C Bond Formation. J. Mol. Catal. B Enzym..

[14] Rajagopalan A., Schober M., Emmerstorfer A., Hammerer L., Migglautsch A., Seisser B., Glueck S.M., Niehaus F., Eck J., Pichler H. (2013). Enzymatic Aerobic Alkene Cleavage Catalyzed by a Mn^3+^-Dependent Proteinase A Homologue. ChemBioChem.

[15] Lara M., Mutti F.G., Glueck S.M., Kroutil W. (2009). Oxidative Enzymatic Alkene Cleavage: Indications for a Nonclassical Enzyme Mechanism. J. Am. Chem. Soc..

[16] Rajagopalan A., Seisser B., Mutti F.G., Schober M., Kroutil W. (2013). Alkene Cleavage by White-Rot *Trametes hirsuta*: Inducing Enzyme Activity by a Fungicide. J. Mol. Catal. B Enzym..

[17] Milovanovic J., Gündüz M.G., Zerva A., Petkovic M., Beskoski V., Thomaidis N.S., Topakas E., Nikodinovic-Runic J. (2021). Synthesis and Laccase-Mediated Oxidation of New Condensed 1,4-Dihydropyridine Derivatives. Catalysts.

[18] Conceição J.C.S., Dias H.J., Peralva C.M.S., Crotti A.E.M., da Rocha Pita S.S., de Oliveira Silva E. (2020). Phenolic Compound Biotransformation by Trametes Versicolor ATCC 200801 and Molecular Docking Studies. Appl. Biochem. Biotechnol..

[19] del Álamo A.C., Pariente M.I., Molina R., Martínez F. (2022). Advanced Bio-Oxidation of Fungal Mixed Cultures Immobilized on Rotating Biological Contactors for the Removal of Pharmaceutical Micropollutants in a Real Hospital Wastewater. J. Hazard. Mater..

[20] Hidayat A., Yanto D.H.Y. (2018). Biodegradation and Metabolic Pathway of Phenanthrene by a New Tropical Fungus, *Trametes hirsuta* D7. J. Environ. Chem. Eng..

[21] del Álamo A.C., Pariente M.I., Vasiliadou I., Padrino B., Puyol D., Molina R., Martínez F. (2018). Removal of Pharmaceutical Compounds from Urban Wastewater by an Advanced Bio-Oxidation Process Based on Fungi Trametes Versicolor Immobilized in a Continuous RBC System. Environ. Sci. Pollut. Res..

[22] Wen P., Wu D., Zheng P., Chen P., Liu S., Fu Y. (2019). Highly Efficient Biosynthesis of Heliotropin by Engineered *Escherichia coli* Coexpressing Trans-Anethole Oxygenase and Formate Dehydrogenase. J. Agric. Food Chem..

[23] Arhipova N., Jansons A., Zaluma A., Gaitnieks T., Vasaitis R. (2015). Bark Stripping of Pinus Contorta Caused by Moose and Deer: Wounding Patterns, Discoloration of Wood, and Associated Fungi. Can. J. For. Res..

[24] Hall T. (1999). BioEdit: A User-Friendly Biological Sequence Alignment Editor and Analysis Program for Windows 95/98/NT. Nucleic Acids Symp. Ser..

[25] Altschul S.F., Gish W., Miller W., Myers E.W., Lipman D.J. (1990). Basic Local Alignment Search Tool. J. Mol. Biol..

[26] Justo A., Hibbett D.S. (2011). Phylogenetic Classification of Trametes (Basidiomycota, Polyporales) Based on a Five-Marker Dataset. Taxon.

[27] Ronquist F., Teslenko M., Van Der Mark P., Ayres D.L., Darling A., Höhna S., Larget B., Liu L., Suchard M.A., Huelsenbeck J.P. (2012). Mrbayes 3.2: Efficient Bayesian Phylogenetic Inference and Model Choice across a Large Model Space. Syst. Biol..

[28] Darriba D., Taboada G.L., Doallo R., Posada D. (2012). JModelTest 2: More Models, New Heuristics and Parallel Computing. Nat. Methods.

[29] Nguyen L.T., Schmidt H.A., Von Haeseler A., Minh B.Q. (2015). IQ-TREE: A Fast and Effective Stochastic Algorithm for Estimating Maximum-Likelihood Phylogenies. Mol. Biol. Evol..

[30] Anisimova M., Gascuel O. (2006). Approximate Likelihood-Ratio Test for Branches: A Fast, Accurate, and Powerful Alternative. Syst. Biol..

