# Supplementary material for: Trametes hirsuta as an Attractive Biocatalyst for the Preparative Scale Biotransformation of Isosafrole into Piperonal
Source: Molecules. 2023 Apr 21;28(8):3643. doi: 10.3390/molecules28083643 (PMC10142777; doi:10.3390/molecules28083643)
Supplement: Supplementary file 1 [file molecules-28-03643-s001.zip › molecules-2310135-supplementary.pdf]

# Supplementary Materials

## *Trametes hirsuta* as an attractive biocatalyst for the preparative scale biotransformation of isosafrole into piperonal

**Dawid Hernik [1,*], Ewa Szczepańska [1], Elisabetta Brenna [2], Katarzyna Patejuk [3], Teresa Olejniczak [1], Tomasz Strzała [4] and Filip Boratyński [1,*]**

[1] Department of Food Chemistry and Biocatalysis, Wroclaw University of Environmental and Life Sciences, Norwida 25, 50-375 Wrocław, Poland; dawid.hernik@upwr.edu.pl (D.H.); ewa.szczepanska@upwr.edu.pl (E.S.), teresa.olejniczak@upwr.edu.pl (T.O.), filip.boratynski@upwr.edu.pl (F.B)

[2] Dipartimento di Chimica, Materiali ed Ingegneria Chimica "Giulio Natta" Politecnico di Milano, Via Mancinelli 7, I-20131 Milan, Italy; mariaelisabetta.brenna@polimi.it (E.B.)

[3] Department of Plant Protection, Wroclaw University of Environmental and Life Sciences, Grunwald Square 24A, 50-363 Wrocław, Poland; katarzyna.patejuk@upwr.edu.pl (K.P.);

[4] Department of Genetics, Wroclaw University of Environmental and Life Sciences, ul. Kozuchowska 7, 51-631 Wrocław, Poland; tomasz.strzala@upwr.edu.pl (T.S.);

* Correspondence: dawid.hernik@upwr.edu.pl (D.H.), filip.boratynski@upwr.edu.pl (F.B.)

Number of pages: 9
Number of figures: 10

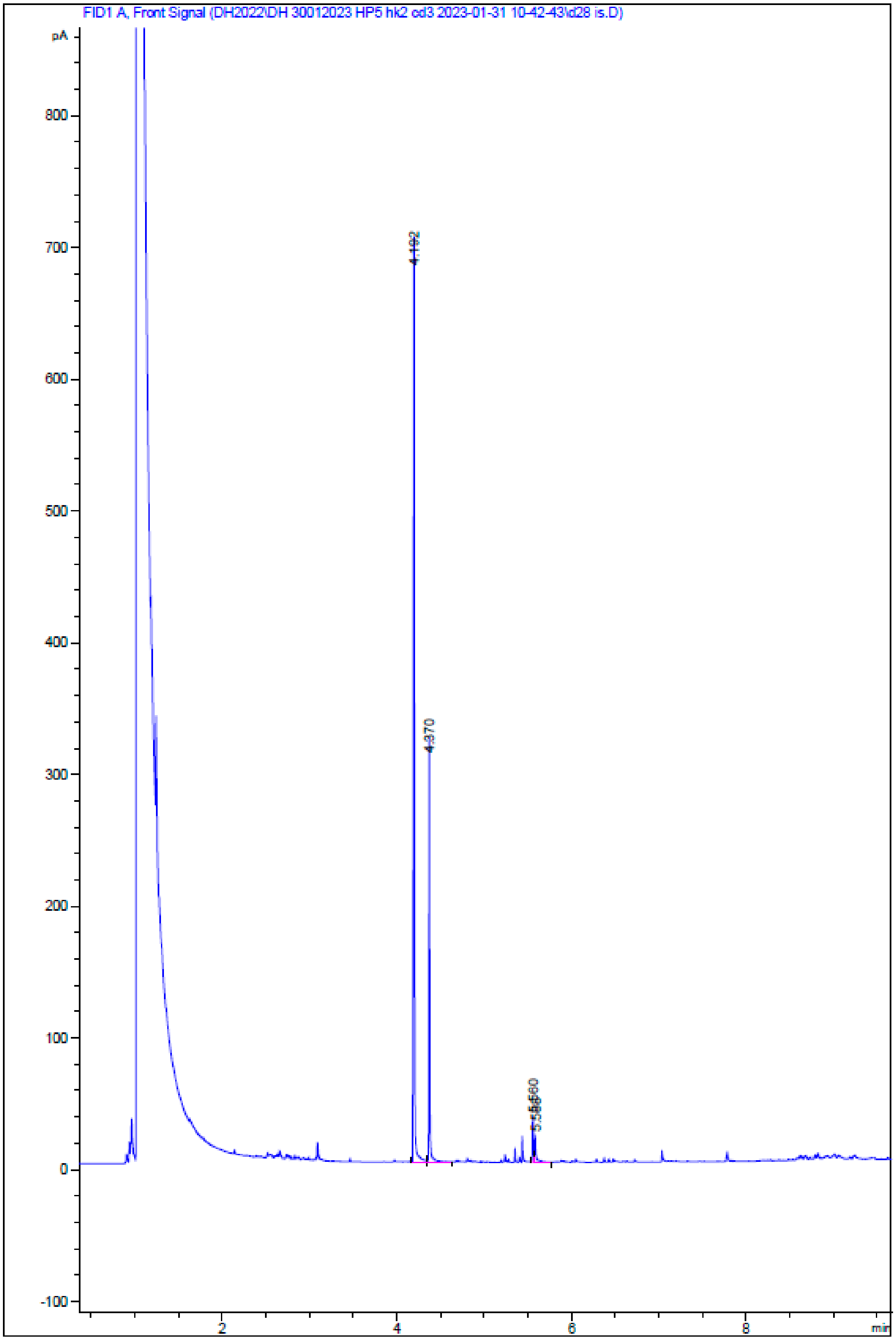


**Figure S1.** Chromatogram of the preparative scale biotransformation of isosafrole (**1a**) to piperonal (**1b**) with *T. hirsuta* d28 after 11 days.

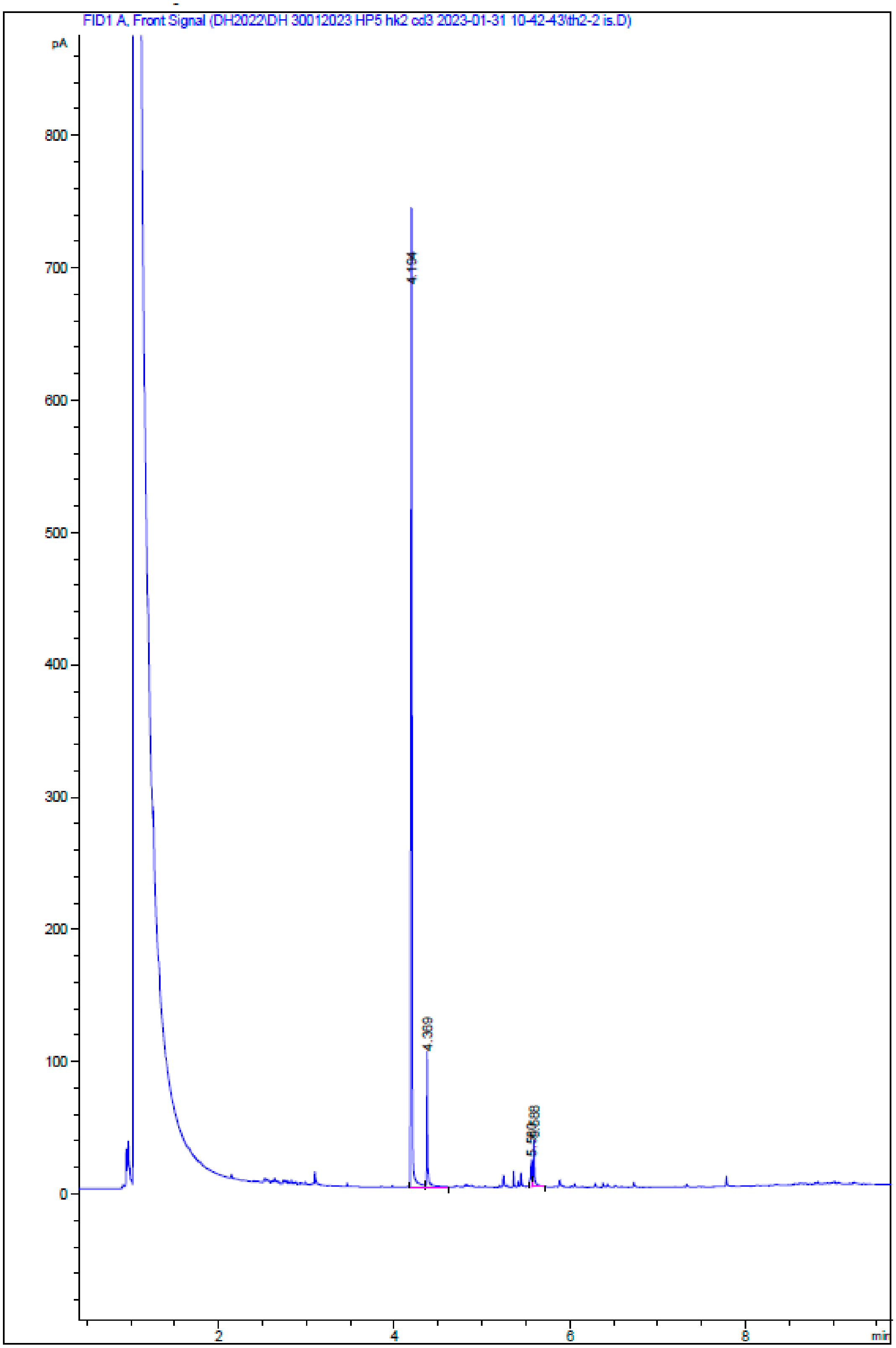


**Figure S2.** Chromatogram of the preparative scale biotransformation of isosafrole (**1a**) to piperonal (**1b**) with *T. hirsuta* TH2_2 after 11 days.

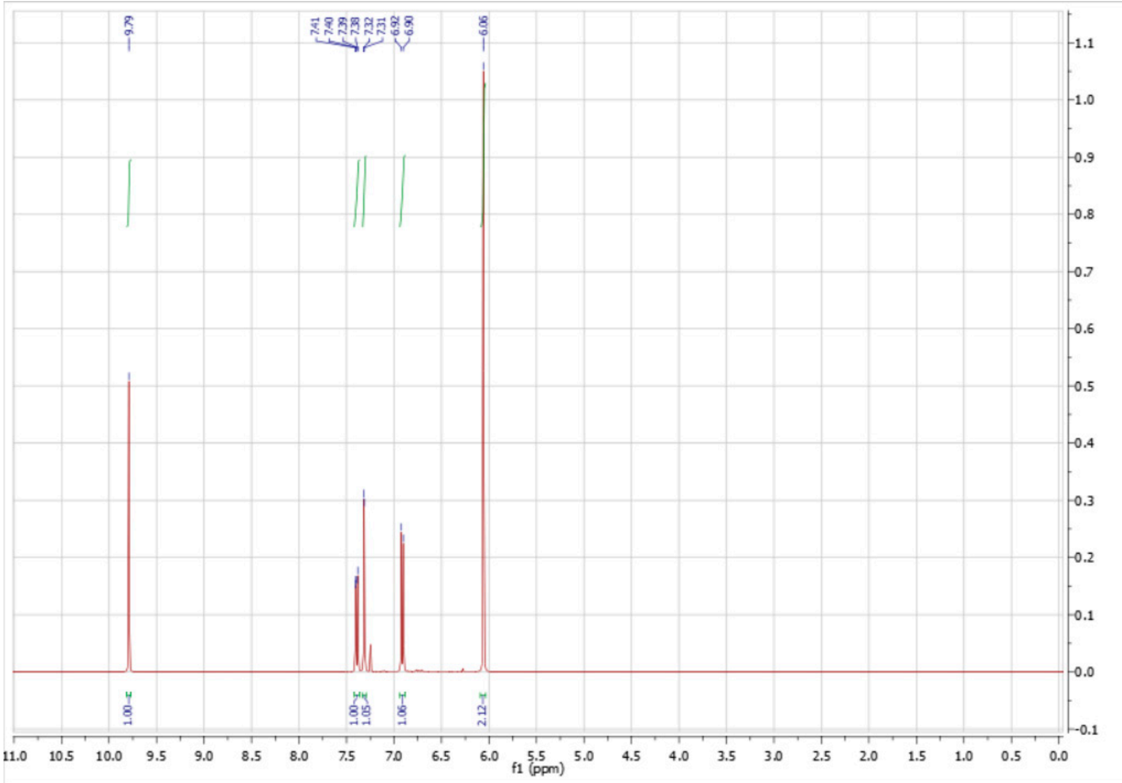


**Figure S3.** H[1] NMR spectrum of piperonal (**1b**).

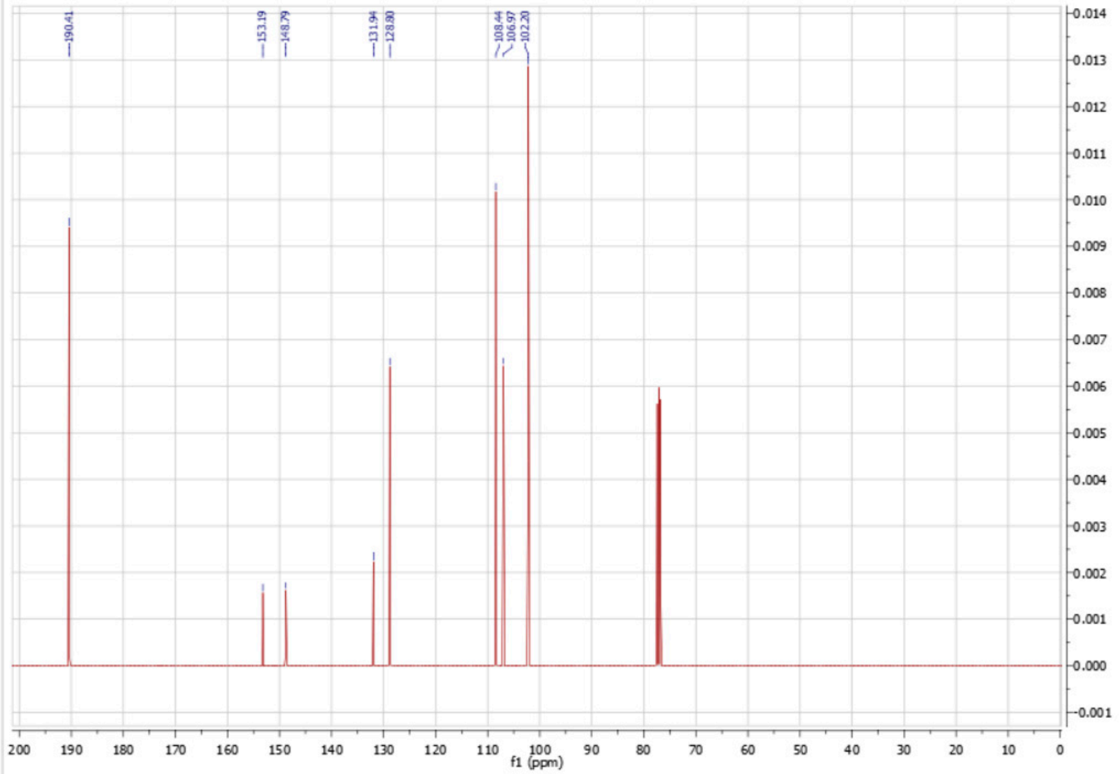


**Figure S4.** C[13] NMR spectrum of piperonal (**1b**).

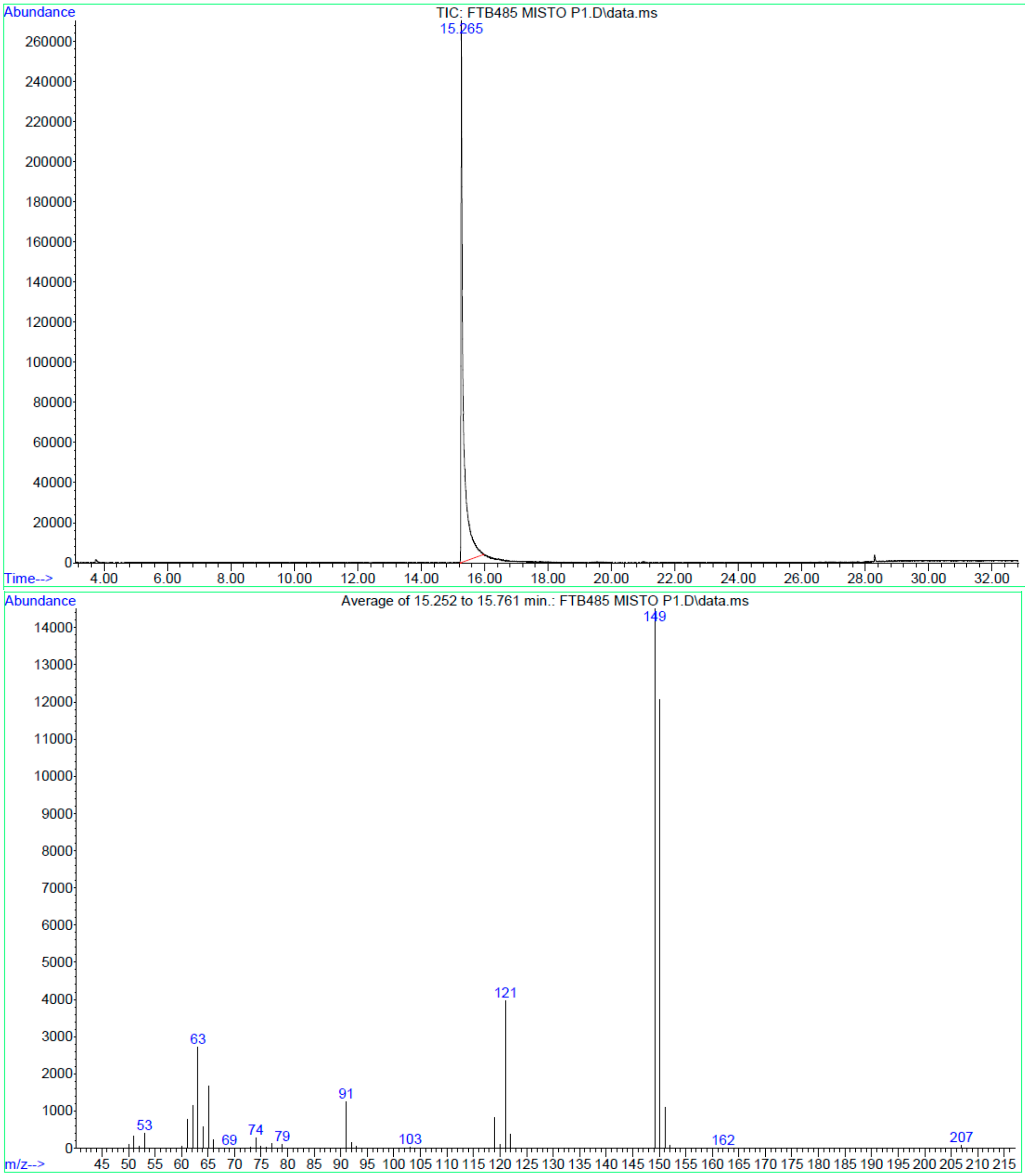


**Figure S5.** GC/MS chromatogram of piperonal (**1b**).

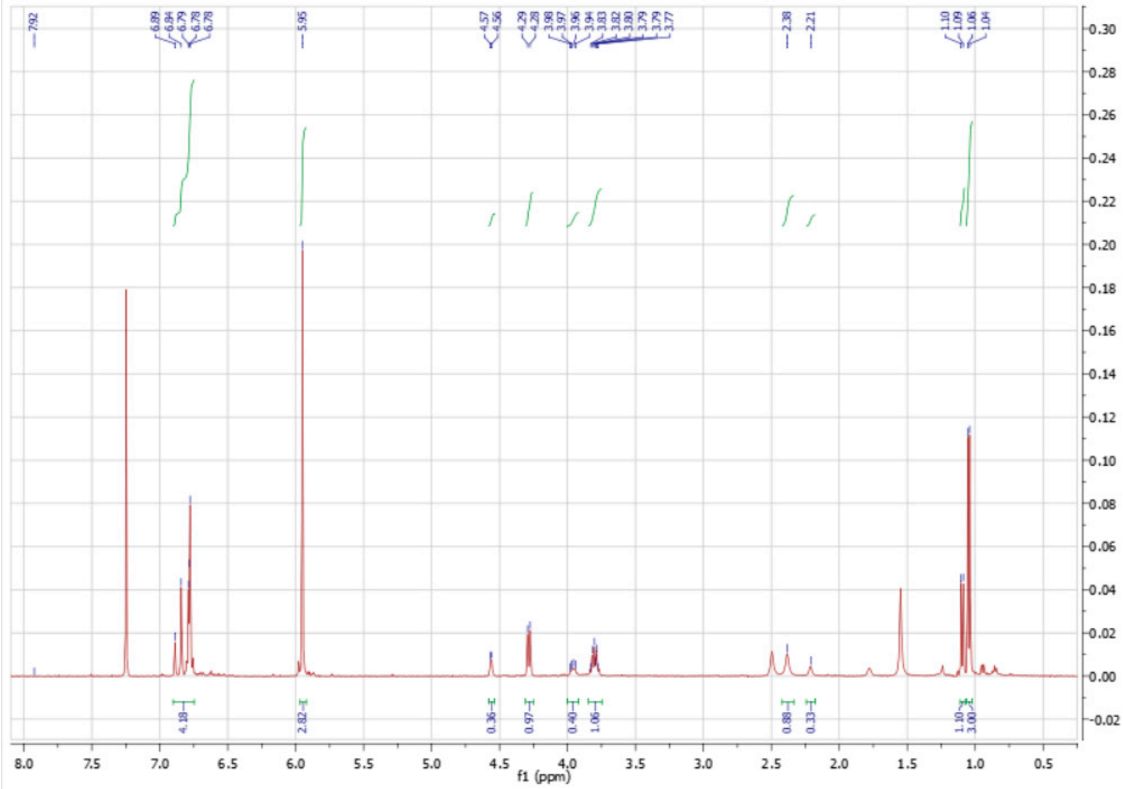

**Figure S6.** H1 NMR spectrum of (1*R**,2*S**) and (1*R**,2*R**)-1-(benzo[1,3]dioxol-5-yl)propane-1,2-diol (**1c**).

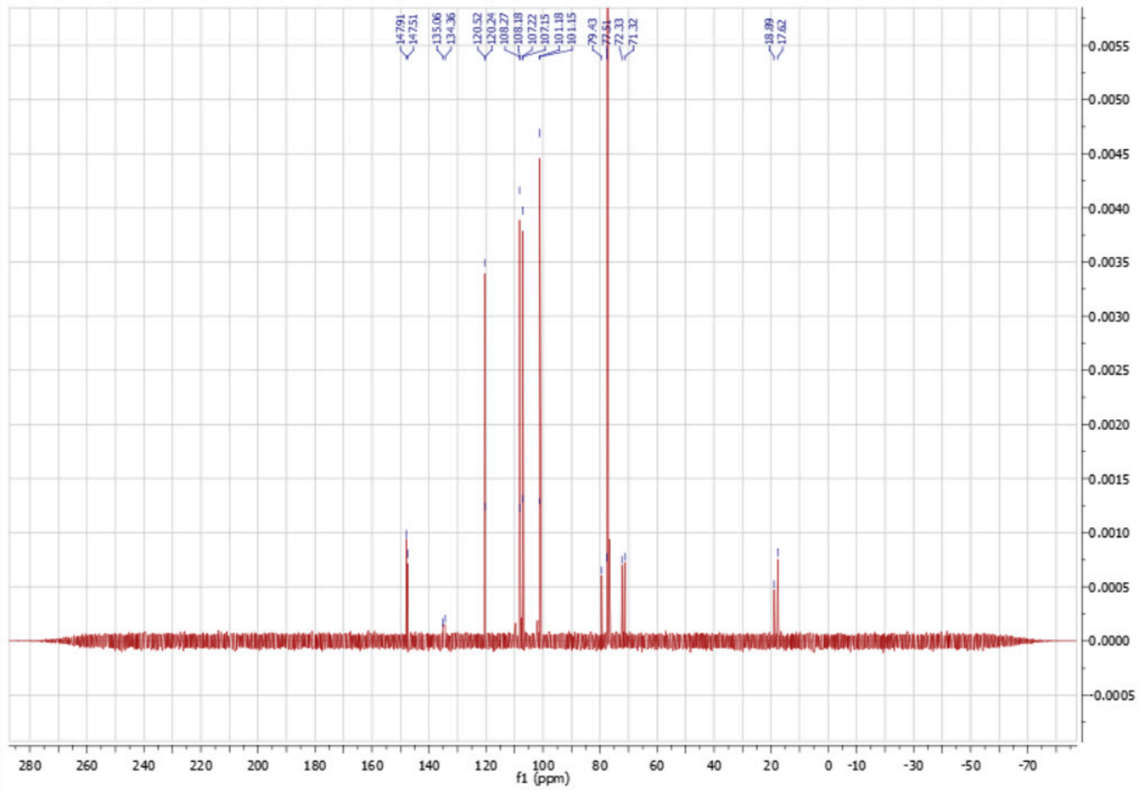

**Figure S7.** C13 NMR spectrum of (1*R**,2*S**) and (1*R**,2*R**)-1-(benzo[1,3]dioxol-5-yl)propane-1,2-diol (**1c**).

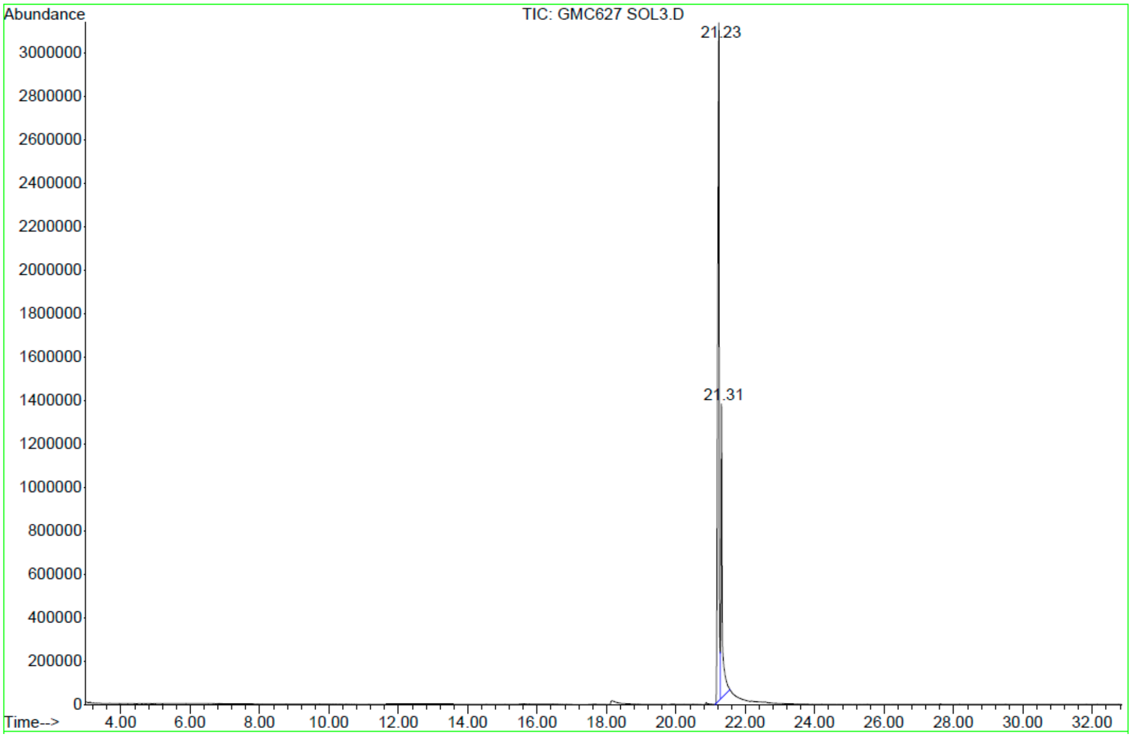


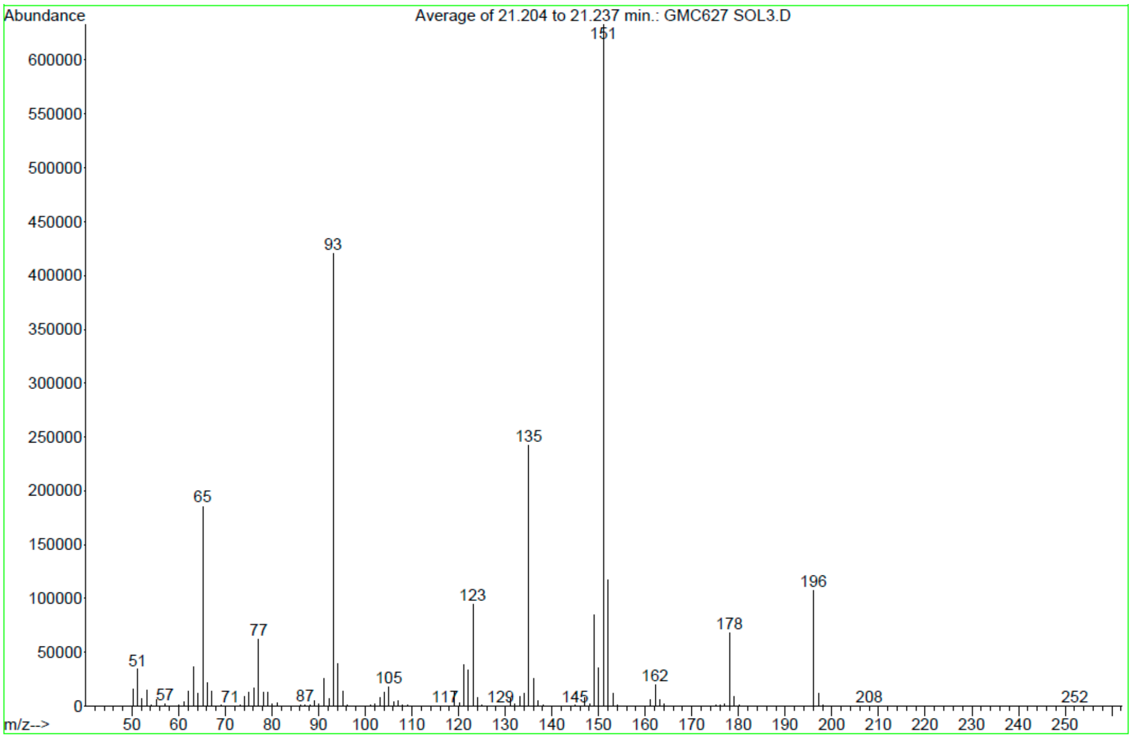


**Figure S8.** GC/MS chromatogram of (1*R**,2*S**) and (1*R**,2*R**)-1-(benzo[1,3]dioxol-5-yl)propane-1,2-diol (**1c**).

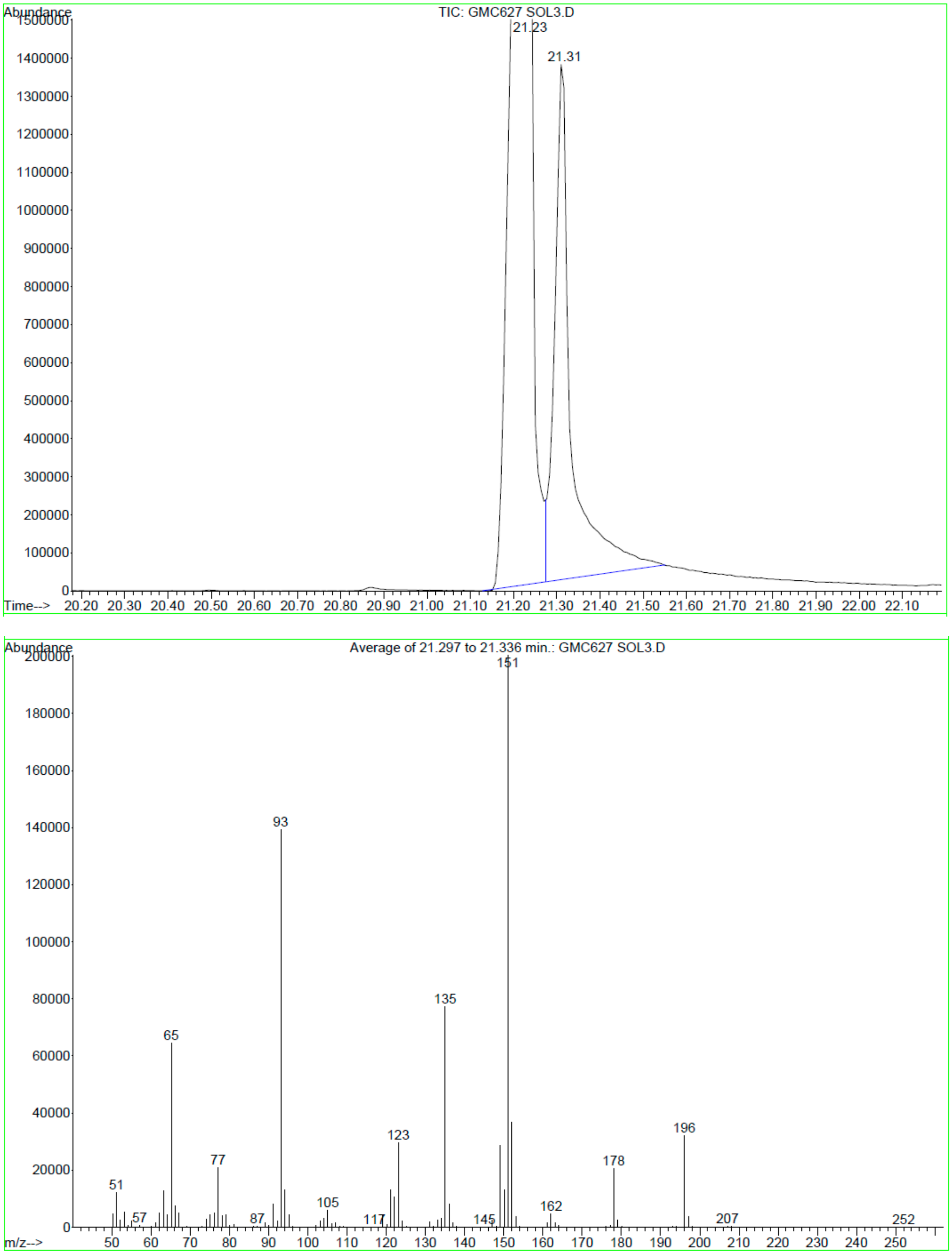


**Figure S9.** GC/MS chromatogram of (1*R**,2*S**) and (1*R**,2*R**)-1-(benzo[1,3]dioxol-5-yl)propane-1,2-diol (**1c**).

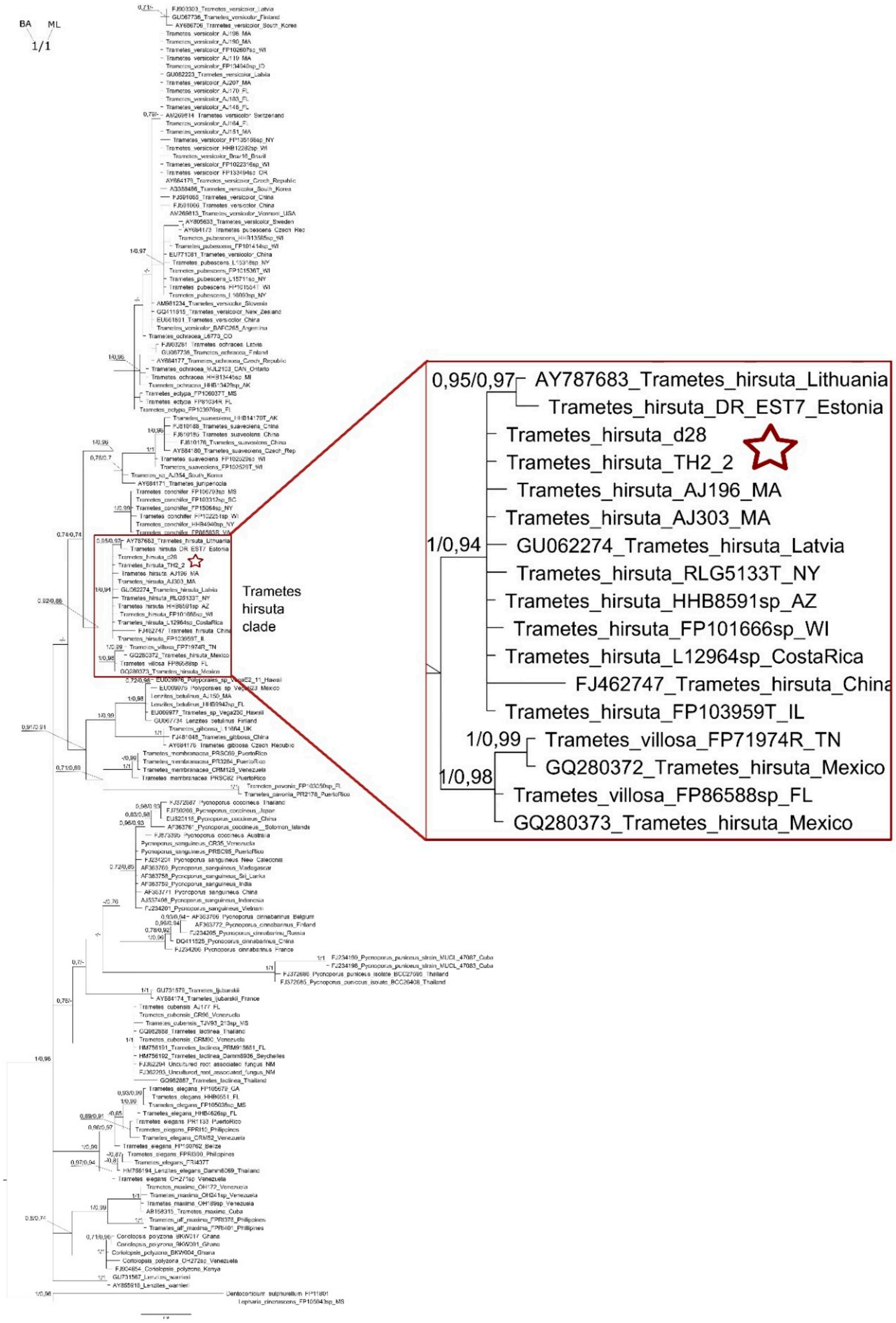


**Figure S10.** Bayesian phylogenetic tree of PolyPeet ITS database sequences of *Trametes* (and other species) along with two sequences from strains d28 and Th2_2 described in this manuscript. Numbers along the nodes are posterior probability of the node (BA) and maximum likelihood bootstrap values (ML) (values below 0,7 were not shown (-)). *Trametes hirsuta* clade was marked with red line and both analysed samples were marked with red star.
